# Onychomycosis due to* Cunninghamella bertholletiae* in an Immunocompetent Male from Central India

**DOI:** 10.1155/2015/703240

**Published:** 2015-11-12

**Authors:** Karuna Tadepalli, Pradeep Kumar Gupta, Dinesh P. Asati, Debasis Biswas

**Affiliations:** ^1^Microbiology Department, AIIMS Bhopal, India; ^2^Dermatology Department, AIIMS Bhopal, India

## Abstract

Onychomycosis is a fungal infection of nails seen frequently in immune competent and immune compromised patients due to dermatophytes,* Candida* spp.,* Fusarium* spp.,* Scopulariopsis brevicaulis*,* Penicillium* spp., and* Aspergillus* spp. We report a case of onychomycosis in a young immunocompetent male who presented onycholysis of a solitary nail without inflammation. The etiological agent was diagnosed to be* Cunninghamella bertholletiae*, a fungus pertaining to the order Mucorales (subdivision Mucoromycotina) and known for some of the invasive lesions among immunocompromised patients. This case demonstrates the association of onychomycosis with* Cunninghamella bertholletiae* in an immune competent individual, not reported so far.

## 1. Introduction

*Cunninghamella bertholletiae* (order Mucorales, subdivision Mucoromycotina) is a saprophytic, ubiquitous fungus found in soil [1]. Although* C. bertholletiae* is known to be the only clinically relevant species in the Cunninghamellaceae family [2–4], other species were recently reported as human pathogens [5]. Although it was first isolated from Brazilian soil samples by Stadel in 1911,* C. bertholletiae* was recently recognized as a cosmopolitan soil organism [6]. The first case of* C. bertholletiae* infection was described in 1958 from a patient with lymph sarcoma and immunosuppressive therapy.* C. bertholletiae* remains a rare cause of mucormycosis and has been described almost exclusively (98%) for immunosuppressed hosts [6]. We report a rare case of onychomycosis due to* C. bertholletiae* in an immune competent patient.

## 2. Case Report

**Figure 1:**
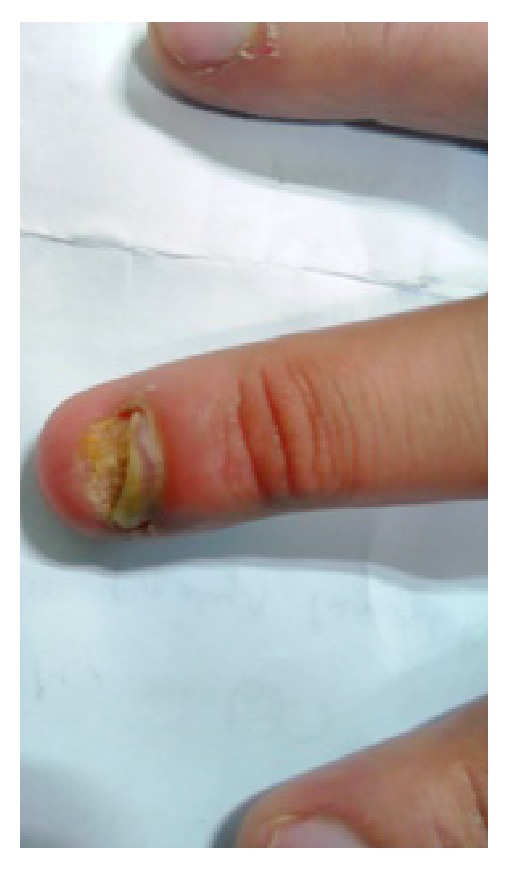
Left middle finger nail showing onycholysis and onychodystrophy.

A 30-year-old male patient residing in Bhopal (Central India) presented to the dermatology department with a lesion in the nail of the left middle finger (Figure 1) for about five years, with history of traumatic injury and no previous use of antifungal therapy. He complained only about the cosmetic aspect as improper growth and unhealthy look of the nail. Professionally, he was involved with mycology laboratory work for few years in United Kingdom and nurtured his hobby of gardening with bare hands very frequently and then returned to India and is continuing with his work.

On local examination he presented onycholysis and onychodystrophy, with no apparent thickening or inflammation of ungueal bed. There was no history of other symptoms except for nail dystrophy. Systemic examination was normal.

**Figure 2:**
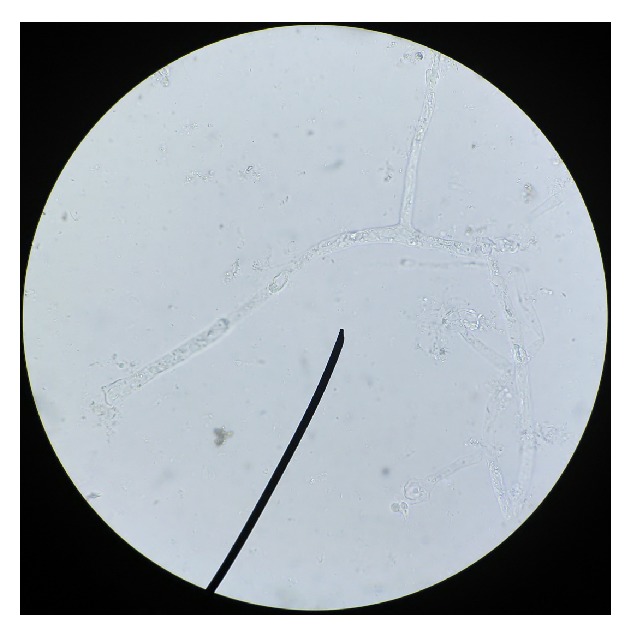
20% KOH wet mount showing broad hyaline sparsely septate hyphae (2-3 septa).

**Figure 3:**
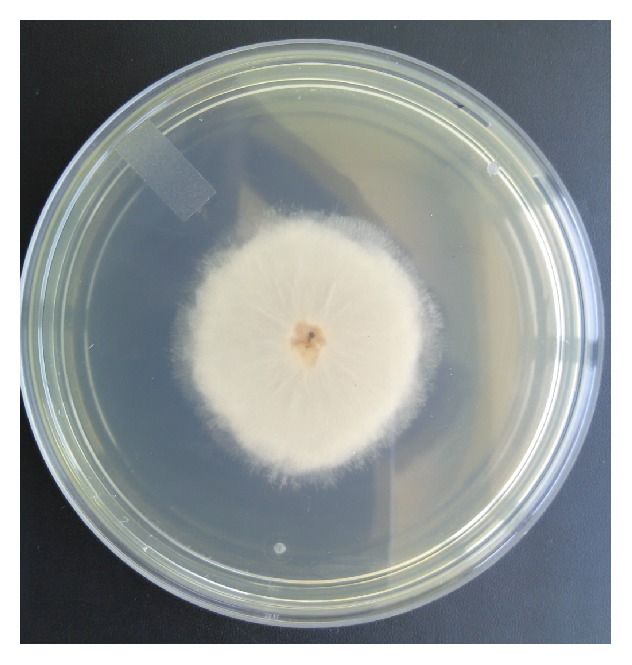
Growth on SDA within 48 hours at 30°C.

**Figure 4:**
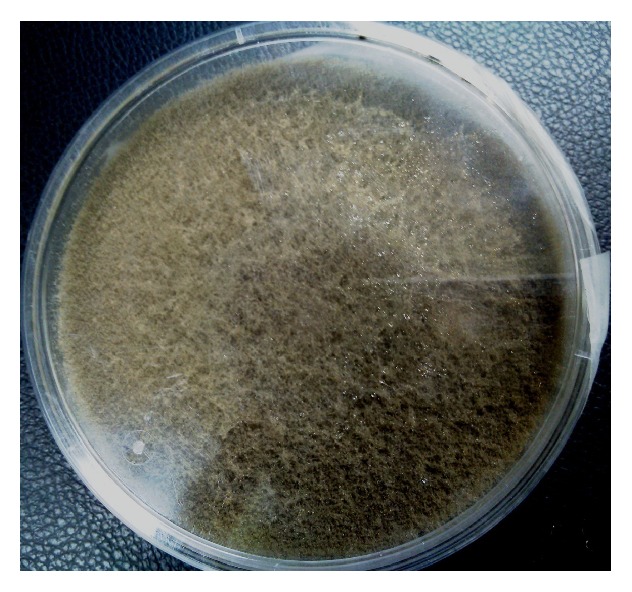
Mature growth on SDA after 72 hours.

**Figure 5:**
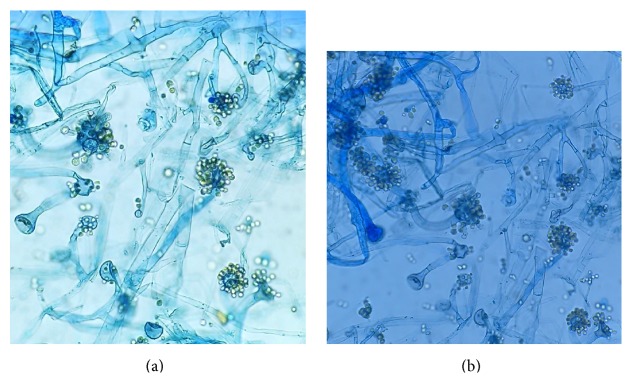
LPCB wet mounts showing branching sporangiophores with vesicles (roughly 32 *μ*m), single-celled sporangiolum and sporangiospores attached to denticles and hyaline broad hyphae with 2-3 septae.

On mycological examination, fungal elements were observed in 20% potassium hydroxide (KOH) preparations as broad hyaline sparsely septate hyphae (Figure 2). Nail specimen was cultured on Sabouraud dextrose agar (SDA) and incubated at 30°C. A rapid growth within 48 hours as wooly white mycelia was observed with reverse pale (Figure 3) and almost filling the whole plate within 72 hours. This mature growth turned grey and further darkened with age, with a reverse remaining pale (Figure 4). On performing lactophenol cotton blue (LPCB) wet mount of the fungus broad, hyaline, and sparsely septate hyphae with branching sporangiophores and terminal vesicle covered with spine-like denticles, and each denticle with a single sporangiolum, later forming each sporangiospore covering the entire surface of the vesicle, was seen. Sporangiospores were spherical to ovoidal (Figures 5(a) and 5(b)).

**Figure 6:**
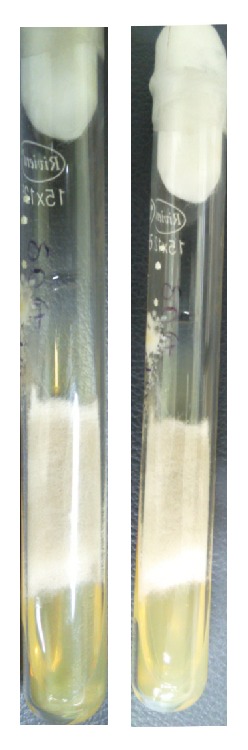
SDA slants incubated at 30°C and 45°C showing similar growth morphology after 48 hours of incubation.

After the first diagnosis, a second sample was taken; the laboratory procedures were repeated. The SDA slants were incubated at 30°C and at 45°C. Both slants showed similar morphology within 3 days of incubation (Figure 6). On repeating LPCB wet mount and microscopic observation similar structural details were seen, suggesting the fungal growth to be* Cunninghamella bertholletiae*.

The patient was advised to apply Ciclopirox topical solution 8% along with oral itraconazole 200 mg/twice a day for one week. Seeing no response the patient was advised for surgical removal of nail, but the patient did not show up for the treatment.

## 3. Discussion

Primary cutaneous and cutaneoarticular cases of mucormycosis caused by* C. bertholletiae* were reported following percutaneous inoculation or trauma in patients with diabetes mellitus, renal transplant recipients, and an i.v. drug abuser with AIDS [6]. Cutaneous* C. bertholletiae* infection was also described for a patient with leukemia who died 18 days after development of a necrotic skin lesion following the use of elastic adhesive tape surrounding a pleural effusion damage site [7]. Cutaneous* C. bertholletiae* infections typically appear as necrotic lesions, with occasional creamy white exudates and granules [6]. There are no reports of onychomycosis documented so far due to* Cunninghamella* spp.

*Cunninghamella* spp. can be found at the lab as environmental contaminants [4]. However, an isolate of* C. bertholletiae* from any clinical material should be analyzed carefully because it is not an ordinary contaminant. In at least two cases, misinterpretation of mycological laboratory findings caused fatal delay or lack of treatment [8, 9]. In our case, presence of fungal elements on direct KOH wet mount could be correlated with the results of the culturing. Also, repeated samples showing the same results suggest the possibility of such saprobic fungal infections particularly in chronic presentations in immunocompetent patients.

*C. bertholletiae* is a fast-growing mold that can grow at room temperature to 45°C.

*C. bertholletiae* grows at temperatures above 40°C, which distinguishes it from* C. elegans*. The species appears as branched sporangiophores terminating at a swollen, terminal vesicle with spherical, ovoidal, or ellipsoidal sporangioles [4, 10–12].

Percutaneous inoculation of* C. bertholletiae*, although less common, has been described from the inpatient, outpatient, and community settings, following pleural taps, peritoneal dialysis, use of blood glucose self-monitoring equipment, and insulin injection [6].

Traumatic injuries have been associated with* C. bertholletiae* infections, including motor vehicle accidents, abrasion occurring while fishing in a patient with leukemia, and nondescript trauma in a human immunodeficiency virus-positive patient [6]. Recently, a rare first case of corneal ulcer caused by* Cunninghamella* spp. following trauma by a stick in an immunocompetent patient has been reported from India [13].

The most common underlying conditions described for patients with* C. bertholletiae* infections are leukemia (51%), diabetes mellitus (19%), nonmalignant haematological diseases (16%), deferoxamine-based therapy (12%), organ transplantation (9%), asplenia (7%), hepatic cirrhosis (2%), AIDS, i.v. drug abuse (2%), and chronic pharmacological immunosuppression for treatment of autoimmune disease (2%) [6].

Only two descriptions of* C. bertholletiae* or* Cunninghamella* species infection have been reported for seemingly immunocompetent patients [9, 14].

Sivakumar et al. from India reported a case of* C. bertholletiae* pulmonary infection in a 42-year-old male patient who developed graft-versus-host disease following bone marrow transplantation [15].

Ours is rare association of* C. bertholletiae* and onychomycosis in an Indian immunocompetent young male; no case is reported so far.

## 4. Conclusion

The laboratory diagnosis of* C. bertholletiae* onychomycosis, as well as with other nondermatophyte fungal infections, should always necessarily be confirmed whether the fungus is the real etiologic agent of the onychomycosis, by collecting repeated fresh samples and processing for KOH wet mount examination and culture for morphological examination, or wherever the facilities are available followed by a final molecular identification by ITS region amplification and sequencing.
